# Social media as a psychoeducative and preventive tool in mental health

**DOI:** 10.1192/j.eurpsy.2022.1475

**Published:** 2022-09-01

**Authors:** R. Molina Ruiz, N. Nuñez Morales, A. González Vazquez

**Affiliations:** 1 Hospital clínico san carlos, Psychiatry, Madrid, Spain; 2 ITA SALUD MENTAL, Psychiatry, Zaragoza, Spain; 3 Hospital Universitario de A Coruña, Psychiatry, A Coruña, Spain

**Keywords:** mental health, social media, prevention, Instagram

## Abstract

**Introduction:**

The use of social networks is an integral part of our daily life as a means of communication. Scientific dissemination through these kind of platforms has expanded enormously in recent years, for example in networks such as Instagram, a free photo, text messaging and video sharing social media application. Instagram has been used extensively in different fields of medicine. Initially it was limited to visually rich fields but it has been extended to others such as mental health. This tool has enormous potential as a means of more effective communication and prevention.

**Objectives:**

To analyze the role of Instagram as a tool of psychoeducation and prevention in mental health

**Methods:**

An analysis of different mental health profiles was carried based on different items: publications, interactions, likes, commentaries, shares, accounts reached, accounts engaged and hashtags.

**Results:**

Mental health has become one of the sanitary fields generating more traffic on social media reaching a great number of publications per day. Topics that generated more interaction were: depression, anxiety, trauma, sleep and emotion regulation difficulties among others. However most mental health information available in social media had not been provided by proffessinals of the mental health but by many others.

**Conclusions:**

Instagram is presented as a valuable mental health prevention tool and professionals should engage and adapt to new scenarios of communication. In the era where information is easy to get but knowledge is difficult to find, experts of mental health should more involved.

**Disclosure:**

No significant relationships.

